# Socioeconomic and geographic determinants of survival of patients with digestive cancer in France

**DOI:** 10.1038/sj.bjc.6603335

**Published:** 2006-09-12

**Authors:** O Dejardin, L Remontet, A M Bouvier, A Danzon, B Trétarre, P Delafosse, F Molinié, N Maarouf, M Velten, E A Sauleau, N Bourdon-Raverdy, P Grosclaude, S Boutreux, G De Pouvourville, G Launoy

**Affiliations:** 1 ‘Cancers & Populations’ ERI 3 INSERM; Faculty of Medicine, Avenue de la Côte de Nacre 14032 Caen Cedex, France; 2 Biostatistic Analyses Unit of ‘Hospices Civils de Lyon’ (HCL), Lyon, France; 3 French Network of Cancer Registries (FRANCIM), Toulouse, France; 4 Centre for Research on Medicine, Science, Health and Society (CERMES/INSERM U750), Villejuif, France

**Keywords:** digestive cancer, road distance, deprivation, distance, multilevel Cox model

## Abstract

Using a multilevel Cox model, the association between socioeconomic and geographical aggregate variables and survival was investigated in 81 268 patients with digestive tract cancer diagnosed in the years 1980–1997 and registered in 12 registries in the French Network of Cancer Registries. This association differed according to cancer site: it was clear for colon (relative risk (RR)=1.10 (1.04–1.16), 1.10 (1.04–1.16) and 1.14 (1.05–1.23), respectively, for distances to nearest reference cancer care centre between 10 and 30, 30 and 50 and more than 90 km, in comparison with distance of less than 10 km; *P*-trend=0.003) and rectal cancer (RR=1.09 (1.03–1.15), RR=1.08 (1.02–1.14) and RR=1.12 (1.05–1.19), respectively, for distances between 10 and 30 km, 30 and 50 km and 50 and 70 km, *P*-trend=0.024) (*n*=28 010 and *n*=18 080, respectively) but was not significant for gall bladder and biliary tract cancer (*n*=2893) or small intestine cancer (*n*=1038). Even though the influence of socioeconomic status on prognosis is modest compared to clinical prognostic factors such as histology or stage at diagnosis, socioeconomic deprivation and distance to nearest cancer centre need to be considered as potential survival predictors in digestive tract cancer.

The incidence of digestive cancers is still increasing, mainly due to colorectal cancer trends (Parkin *et al*, 2002), and retains a poor prognosis, as reported by the European Cancer Registries (EUROCARE) study (Berrino *et al*, 2003). This study found a large range of digestive cancer survival within Europe, while a survival difference in colorectal cancer between Europe and the USA has also been reported (Ciccolallo *et al*, 2005). To explain such inter-country differences, besides the important effect of stage at diagnosis and surgery, the influence of health system organisation and social determinants needs to be further investigated.

The 5-year relative survival of the 60 000 cases of digestive cancer each year in France (Remontet *et al*, 2003) is reasonably high compared to the European mean. Nevertheless, a few French studies of colorectal cancer from a single cancer registry (Launoy *et al*, 1992; Desoubeaux *et al*, 1997) have suggested social inequalities in survival, underprivileged patients having poorer prognosis than more affluent patients. We recently suggested that access to specialised care was strongly associated with road distance to the nearest specialised centre especially for women and elderly patients (Dejardin *et al*, 2005). Recent research in different health-care systems also suggests a relationship between surgical volume and survival (Kee *et al*, 1999; Shrag *et al*, 2000; Platell *et al*, 2003). Geographical access to reference centres is therefore relevant to the study of social inequalities in cancer survival.

This study, based on data on all digestive cancers registered by the French Network of Cancer Registries (FRANCIM) between 1980 and 1997 in France, investigates the influence of the socioeconomic and geographical environment on survival of affected patients.

## MATERIALS AND METHODS

### Population and data

The study population comprised 81 268 patients with digestive tract cancer registered between 1 January 1980 and 31 December 1997 in 12 cancer registries (Calvados, Côte d'Or, Doubs, Hérault, Isère, Loire-Atlantique, Manche, Bas-Rhin, Haut-Rhin, Saône et Loire, Somme and Tarn), all belonging to the French Network of Cancer Registries (FRANCIM); as such, their exhaustiveness and data quality are regularly checked by the French Institute of Health and Medical Research (INSERM) and the International Agency for Research on Cancer (IARC). General characteristics of the study population, representing 14.4% of the French population (*n*=8 441 366), are detailed in Table 1.

Survival was defined as the interval in months between diagnosis and last information on vital status. Two types of variable were used in the study, individual and aggregate.

Level-1 individual variables: data on age, gender, cancer site and *Communes* (the smallest administrative district in France under the government of a mayor and a municipal council=*town/village*) of residence were individually known for all patients and thus considered as individual variables. Cancer site was classified according to ICDO third edition: oesophagus C15-C15.9, stomach C16-C16.9, small intestine C17-C17.9, colon C18-C18.9, rectum (including rectosigmoid junction) C19.9-C21.8, liver C22-C22.1, gall bladder and biliary tract C23.9-C24.9 and pancreas C25-C25.9.

Level-2 aggregate variables: to compensate for the lack of individual information on socioeconomic status, each patient was attributed the socioeconomic characteristics of his neighbourhood (*Commune* level) using French Census data provided by the INSEE (1999) – *National Institute for Statistics and Economic Studies*, that is, proportion of people who did not complete secondary education to age 15, mean taxable income, unemployment rate, proportion of housing without bath or shower, manual worker proportion. Mean taxable income (2003) of each *Commune* was obtained from the ‘Direction Générale des Impôts’, the tax department.

French public health authorities have defined reference care centres as centres capable of providing care management of serious pathologies with bad prognosis as well as rare pathologies. In our study, the reference care centres are mainly composed of a university hospital and a specialised cancer centre. Road distance to nearest cancer reference care centre (always located in regional capitals) was calculated using MAPINFO 6.5 (MapInfo corporation) and CHRONOMAP (Magellan engineering). Road database (IGN route 500) of the National Geographic Institute (IGN) included 500 000 km of road. These variables were also considered as level-2 variables.

The population study was distributed within 5416 of the 6289 *Commune*s of the 12 French counties (86.11%). The mean *Commune* population was 1342.1 (range 270 343–6). The mean number of cases per *Commune* was 15.00.

### Analysis

A multilevel Cox proportional hazards model was used to take into consideration the hierarchical data structure (Goldstein, 1995). Level 1 included patient characteristics (age, sex and year of diagnosis), level 2 was composed of the *Commune*'s social indicator. All models were adjusted on department of residence (level 3).

Iterative general least square (IGLS) (Poisson first order marginal quasi likelihood (MQL)) was used to estimate the model. For each cancer site, we investigated the influence of each of six socioeconomic level-2 variables after adjustment on age, gender, year at diagnosis and department of residence in eight separate models. The influence of social variables on survival was investigated using linear trend for categorised variables.

Mlwin 2.01 (multilevel model project, London Institute of Education) with survival macro was used to perform the multilevel Cox model.

## RESULTS

Table 2 shows the influence of socioeconomic and geographical environment on survival for patients with digestive tract cancer. The association between socioeconomic and geographical aggregate variables and survival differed by cancer site. This association was clear for colon and rectal cancer (*n*=28 010 and *n*=18 080, respectively) but not for gall bladder and biliary tract (*n*=2893) or small intestine (*n*=1038) cancers. For oesophageal, stomach, liver and pancreatic cancers, prognosis was associated with only one or two socioeconomic or geographic variables. Each of the socioeconomic aggregate variables was associated with at least one cancer site, the most frequently with distance to the nearest cancer reference care centre (hereafter ‘cancer centre’).

### Oesophageal cancer

Distance to the nearest cancer centre was not associated with survival. The proportion of housing without bath or shower was significantly associated with prognosis (*P*-trend=0.004). In comparison with patients living in a *Commune* with less than 1.1% houses without bath or shower, patients living in a *Commune* with a high proportion of housing without bath or shower had poorer prognosis (relative risk (RR)=1.08 (1.00–1.16) and RR=1.12 (1.04–1.20), respectively for 1.7–2.7 and >2.7% of housing without bath or shower).

### Stomach cancer

Distance to nearest cancer centre was associated with prognosis (*P*-trend=0.0014) even after adjustment for age, gender and department of residence, patients living far from a cancer centre having poorer prognosis than those living closer (RR=1.09 (1.00–1.20) and RR=1.11 (1.00–1.23), respectively, for 50–70, and >90 km).

The association of prognosis with the proportion of housing without bath or shower was close to significant (*P*=0.08), being significant when the department of residence was excluded from the analysis; those in a *Commune* with a high proportion of housing without bath or shower having poorer prognosis (*P*-trend <0.001) (results not shown).

### Small intestine

Although the prognosis of cancer did not decrease with the distance to nearest cancer centre, the prognosis of patients living 30–50 km from a cancer centre was significantly poorer than those living less than 10 km away (RR=1.48 (1.18–1.85)). An increasing proportion of manual workers was somewhat associated with poorer prognosis (*P*-trend=0.072), becoming significant when the proportion of manual workers was used as a continuous variable (*P*-trend=0.02) (results not shown).

### Colon cancer

After adjustment for individual characteristics, patients living far from a cancer centre had poorer survival than those living closer (RR=1.10 (1.04–1.16), 1.10 (1.04–1.16) and 1.14 (1.05–1.23), respectively, for distance of 10–30 km, 30–50 km and over 90 km, compared to less than 10 km, *P*=0.003).

Compared to patients living in a *Commune* with less than 14% of people who did not complete secondary education to age 15, patients living in a *Commune* with a greater proportion had a poorer survival (*P*-trend=0.002). Patients living a *Commune* with low mean taxable income (<13548 euros) had poorer survival than those in a more affluent *Commune* (RR=0.92 (0.88–0.97) with mean taxable income >16 711 euros) (*P*-trend=0.002).

### Rectum cancer

Patients living far from a cancer centre had poorer prognosis than those living closer (RR=1.09 (1.03–1.15), RR=1.08 (1.02–1.14) and RR=1.12(1.05–1.19), respectively, for distances to nearest cancer centre of 10–30, 30–50 and 50–70 km compared with less than 10 km, *P*-trend=0.024). In comparison with patients living in a *Commune* with a low proportion of people who did not complete secondary education to age 15, those living in a *Commune* with a higher proportion of people who did not complete secondary education to age 15 had a poorer survival (RR=1.07 (1.02–1.13) and RR=1.09 (1.03–1.15), respectively, for proportion of people who did not complete secondary education to age 15 of 19–23 and >23%). In comparison with patients living in a *Commune* with less than 13% of manual workers, those living in a *Commune* with a higher proportion have poorer survival (RR=1.07 (1.01–1.13), RR=1.10 (1.04–1.16) and RR=1.07 (1.01–1.14), respectively for proportion of manual workers between 13 and 17, 17 and 21 and >21%; *P*-trend=0.009).

### Liver

Distance to the nearest cancer centre was not associated with prognosis of liver cancer. Patients living in a *Commune* with a high unemployment rate had poorer survival than those in a less-deprived *Commune* (RR=1.09 (1.01–1.18) and RR=1.09 (1.00–1.19), respectively, for unemployment rate 12–15% and above 15%, compared with below 8%; *P*-trend=0.010).

### Gall bladder and Biliary tract

Neither socioeconomic variables nor distance to the nearest cancer centre were significantly associated with prognosis of gall bladder or biliary cancer.

### Pancreas

Distance to the nearest cancer centre was not significantly associated with prognosis. Patients living in a *Commune* with a high proportion of manual workers had poorer prognosis than those living in a more affluent *Commune* (*P*-trend=0.032). The association with unemployment rate was suggestive (*P*-trend=0.073) and became significant (*P*=0.034) when tested using a continuous variable.

## DISCUSSION

These results support the hypothesis that socioeconomic and geographical environment influences prognosis of digestive cancer in France, as previously found in other European countries and in the USA (Woods *et al*, 2006). This influence appears to be significant for the most common cancers with better prognosis (colon, rectum), and to a lesser extent for cancers with poorer prognosis (oesophagus, stomach, liver and pancreas). No influence was found for gall bladder and biliary tract cancer.

Certain methodological aspects of our study are relevant. In the absence of individual-based social information, the surrogate of an aggregate (*Commune* level) social indicator was used. Moreover, no deprivation index is available in France, prompting a multidimensional approach to poverty (remote patient; education; income; working conditions and accommodation).

The level of geographical unit used in our study was the *Commune*. *Commune*s are the smallest administrative unit in France but their population sizes vary widely. The National Institute for Statistics and Economic Studies (INSEE) has defined a smaller unit: ‘Ilots regroupés d'indice statistique’ (IRIS). Regional capital and other important *Commune*s are divided into several IRIS and small *Commune*s constitute one IRIS. Therefore, the use of IRIS increases the accuracy of socioeconomic variables in such studies (Woods *et al*, 2005). Unfortunately, the lack of information on patients' addresses prevented us from using the IRIS in our study. Socioeconomic inequalities highlighted in our study are likely to have been increased had we used the IRIS unit.

The use of relative survival, instead of crude survival, would have enabled us to adjust for comorbidity associated with age and gender, but to our knowledge, no multilevel relative survival model is available. As recently stressed (Auvinen and Karjalainen, 1997), relative survival tends to underestimate social inequalities in survival so we have preferred multilevel analysis rather than specific mortality.

The present study suffers from the lack of information on change in Census data during the study period. In fact, aggregate variables were collected in 1999 by the French Census and in 2003 for income tax purposes in an annual survey by the Direction Générale des Impôts, the tax department. The National Institute for Statistics and Economic Studies does not organise a Census each year. Furthermore, since multiple comparisons were made (six factors were tested for each of the eight cancers), some tests (two or three) may be significant by chance.

Nevertheless, our results accord with previous studies that stressed the appreciable gap between the most deprived and the most affluent in survival (Woods *et al*, 2006). At least in England and Wales, this was more widespread in the 1990s than previously (Coleman *et al*, 2004). In France, two studies, based on small samples, demonstrated that *Communes* with a high proportion of dwellings without bath or shower were negatively correlated with cancer survival (Monnet *et al*, 1993) and that farmers of both sexes and unemployed men had poorer survival than other socioeconomic categories (Desoubeaux *et al*, 1997). The frequent association of this housing measure suggested that it was a relevant socioeconomic indicator.

For cancers with good prognosis, in our study, the influence of socioeconomic aggregate variables on survival were similar to those in a Norwegian population-based study also using multilevel survival (discrete-time hazard regression) (Kravdal, 2006). In the Norwegian study, education variables (at individual and community level) were the most relevant socioeconomic indicators.

In our study, unlike the Norwegian study (Kravdal, 2006), road distance between cancer centres and the *Commune* of residence is a predictor of cancer survival for almost half of the cancer sites. This contradiction between the two studies may be partially explained by the adjustment for cancer stage at diagnosis in the Norwegian study. Lack of information on stage at diagnosis in our study restricts the interpretation of our results. Cancer stage at diagnosis is often more advanced in deprived people (Ionescu *et al*, 1998; Ciconne *et al*, 2000), although one recent study (Brewster *et al*, 2001) failed to find this for colorectal cancer using Carstairs deprivation categories. The explanation of social inequalities in cancer survival by the difference in stage at diagnosis is attractive as a consequence of the importance of stage at diagnosis as a prognostic factor. Nevertheless, as underlined by a recent review (Woods *et al*, 2006), stage at diagnosis does not entirely account for the gap in cancer survival between the most deprived and the most affluent.

Although geographic and socioeconomic effects on prognosis are modest compared to clinical prognostic factors such as histology or stage at diagnosis, they warrant consideration as potential survival predictors in digestive tract cancer. Few such studies have been carried out, partly due to the lack of individual-based information. Our study implies that the French health-care system, which theoretically provides free access to care to all patients, with extensive hospital services throughout the country, does not prevent social inequalities in cancer management.

## Figures and Tables

**Table 1:** Digestive cancer diagnosed between 1980 and 1997 in 12 French Communes

		***N*=81 268**
	***N***	**%**
Level 1: patient characteristics		
*Age (years)*		
<61	24 197	29.77
61–70	11 910	14.66
70–78	13 006	16.00
>78	32 155	39.57
		
*Gender*		
Men	48 741	59.98
Women	32 527	40.02
		
*Year of diagnosis*		
1980	1653	2.03
1981	1690	2.08
1982	2284	2.81
1983	2424	2.98
1984	2450	3.01
1985	2436	3.00
1986	2519	3.10
1987	2554	3.14
1988	2516	3.10
1989	5400	6.64
1990	5541	6.82
1991	5990	7.37
1992	6331	7.79
1993	6405	7.88
1994	6976	8.58
1995	7892	9.71
1996	8039	9.89
1997	8168	10.05
		
*Site*		
Oesophagus	7813	9.61
Stomach	11 619	14.30
Colon	28 010	34.47
Rectum	18 080	22.25
Small intestine bowel	1038	1.28
Liver	5797	7.13
Gall bladder	2893	3.56
Pancreas	6018	7.41
		
*Departments* a		
Calvados (1980–1997)	10 169	12.51
Côte d'Or (1980–1997)	8489	10.45
Doubs (1989–1997)	3705	4.56
Hérault (1995–1997)	2418	2.98
Isère (1989–1997)	8423	10.36
Loire Atlantique (1991–1997)^b^	3885	4.78
Manche (1996–1997)	2098	2.58
Bas-Rhin (1980–1997)	17 122	21.07
Haut-Rhin (1989–1997)	6781	8.34
Saône et Loire (1982–1997)	10 410	12.81
Somme (1989–1997)	4418	5.44
Tarn (1989–1997)	3350	4.12
		
*Last known status (at 1 January 2002)*		
Censored	15.229	18.74
Death	61.820	76.07
Lost of follow up	4.243	5.19
		
Level 2: Commune characteristics		
*Distance to nearest reference cancer care center (km)*		
<10	21 621	26.60
10–30	13 924	17.13
30–50	13 816	17.00
50–70	12 569	15.47
70–90	9235	11.36
>90	10 103	12.43

a Period contributions are shown in brackets.

b Colorectal cancer only.

**Table 2:** Geographical and socioeconomic environment influence on survival for patient with digestive tract cancer (models adjusted for age, gender, year of diagnosis and department)

	**Oesophageal cancer (*N*=7813)**				**Stomach cancer (*N*=11 619)**				**Small intestine cancer (*N*=1038)**				**Colon cancer (*N*=28010)**			
	**RR**	**Confidence interval^a^**		***P*-trend**	**RR**	**Confidence interval^a^**		***P*-trend**	**RR**	**Confidence interval^a^**		***P*-trend**	**RR**	**Confidence interval^a^**		***P*-trend**
*Distance to nearest cancer reference care centre (km)*																
<10	1.00			0.335	1.00			0.014	1.00			0.107	1.00			0.003
10–30	1.04	0.97	1.12		1.00	0.93	1.06		0.89	0.70	1.13		1.10	1.04	1.15	
30–50	1.03	0.96	1.11		1.06	1.00	1.13		1.48	1.18	1.85		1.10	1.04	1.15	
50–70	1.03	0.95	1.12		1.05	0.97	1.13		1.11	0.82	1.51		1.06	1.00	1.12	
70–90	1.04	0.93	1.16		1.09	1.00	1.20		1.33	0.94	1.90		1.09	1.01	1.17	
>90	1.06	0.93	1.22		1.11	1.00	1.23		0.94	0.63	1.39		1.14	1.05	1.24	
																
*Proportion who did not complete secondary education to age 15 (%)*																
<14	1.00			0.261	1.00			0.138	1.00			0.122	1.00			0.002
14–19	1.03	0.96	1.11		1.03	0.97	1.10		1.21	0.97	1.52		1.08	1.03	1.13	
19–23	1.05	0.98	1.13		1.01	0.95	1.08		1.18	0.94	1.48		1.08	1.03	1.13	
>23	1.04	0.97	1.12		1.06	0.99	1.12		1.23	0.98	1.54		1.08	1.03	1.14	
																
*Mean taxable income (in euros)*																
<13 458	1.00			0.133	1.00			0.342	1.00			0.975	1.00			0.003
13 458–14 997	1.00	0.94	1.07		0.97	0.92	1.03		0.96	0.77	1.20		0.97	0.92	1.01	
14 997–16 711	0.96	0.89	1.03		0.96	0.90	1.02		1.18	0.93	1.50		0.99	0.94	1.04	
>16 711	0.95	0.89	1.02		0.97	0.93	1.01		0.96	0.76	1.23		0.92	0.88	0.97	
																
*Unemployment rate (%)*																
<7.9	1.00			0.538	1.00			0.591	1.00			0.547	1.00			0.865
7.9–12	0.96	0.90	1.03		0.93	0.88	0.98		0.91	0.74	1.13		1.00	0.96	1.05	
12–15	0.99	0.92	1.06		0.94	0.88	0.99		1.03	0.83	1.27		1.00	0.95	1.05	
>15	1.02	0.94	1.10		0.98	0.92	1.05		1.03	0.82	1.30		1.00	0.94	1.05	
																
*Proportion of houses without bath or shower (%)*																
<1.1	1.00			0.004	1.00			0.080	1.00			0.823	1.00			0.143
1.1–1.7	1.06	0.98	1.14		1.02	0.96	1.08		1.10	0.88	1.36		1.06	1.01	1.11	
1.7–2.7	1.08	1.00	1.16		1.02	0.96	1.08		1.13	0.92	1.40		1.07	1.02	1.12	
>2.7	1.12	1.04	1.20		1.06	1.00	1.13		1.01	0.80	1.26		1.03	0.98	1.09	
																
*Proportion of manual workers (%)*																
<13	1.00			0.142	1.00			0.271	1.00			0.072	1.00			0.153
13–17	0.93	0.87	1.00		0.99	0.93	1.05		1.03	0.99	1.07		1.02	0.97	1.07	
17–21	0.96	0.89	1.03		0.98	0.92	1.04		1.07	0.85	1.34		1.04	0.99	1.09	
>21	0.93	0.87	1.00		1.04	0.97	1.10		1.23	0.97	1.55		1.03	0.98	1.09	
																

	**Rectum cancer (*N*=18 080)**				**Liver cancer (*N*=5797)**				**Gall bladder and biliary tract cancer (*N*=2893)**				**Pancreatic cancer (*N*=6018)**			
	**RR**	**Confidence interval^a^**		***P*-trend**	**RR**	**Confidence interval^a^**		***P*-trend**	**RR**	**Confidence interval^a^**		***P*-trend**	**RR**	**Confidence interval^a^**		***P*-trend**
*Distance to nearest cancer care centre (km)*																
<10	1.00			0.024	1.00			0.629	1.00			0.529	1.00			0.246
10–30	1.09	1.03	1.15		0.96	0.88	1.04		0.95	0.84	1.07		1.03	0.95	1.13	
30–50	1.08	1.02	1.14		0.98	0.91	1.07		1.02	0.90	1.15		1.05	0.96	1.14	
50–70	1.12	1.05	1.19		0.91	0.83	1.01		1.02	0.88	1.19		1.03	0.93	1.14	
70–90	1.04	0.96	1.13		1.03	0.90	1.17		0.98	0.82	1.17		1.03	0.91	1.17	
>90	1.06	0.96	1.16		1.01	0.87	1.17		1.09	0.89	1.34		1.11	0.96	1.29	
																
*Proportion who did not complete secondary education to age 15 (%)*																
<14	1.00			0.002	1.00			0.444	1.00			0.899	1.00			0.197
14–19	1.05	0.99	1.11		0.95	0.87	1.03		1.03	0.91	1.16		1.04	0.96	1.13	
19–23	1.07	1.02	1.13		0.94	0.87	1.02		1.03	0.92	1.16		1.07	0.99	1.16	
>23	1.09	1.03	1.15		0.97	0.89	1.05		0.99	0.88	1.12		1.05	0.97	1.13	
																
*Mean taxable income (in euros)*																
<13 458	1.00			0.132	1.00			0.637	1.00			0.991	1.00			0.167
13 458–14 997	0.97	0.92	1.02		1.01	0.93	1.09		1.03	0.91	1.16		1.06	0.98	1.14	
14 997–16 711	0.96	0.91	1.02		0.99	0.90	1.07		1.00	0.88	1.13		1.03	0.95	1.12	
>16 711	0.96	0.90	1.01		0.99	0.91	1.07		0.99	0.87	1.12		0.95	0.88	1.03	
																
*Unemployment rate (%)*																
<7.9	1.00			0.990	1.00			0.010	1.00			0.744	1.00			0.073
7.9–12	1.03	0.98	1.09		1.02	0.94	1.10		1.03	0.92	1.15		1.06	0.99	1.14	
12–15	1.01	0.96	1.06		1.09	1.01	1.18		1.02	0.92	1.14		1.05	0.95	1.16	
>15	1.01	0.95	1.07		1.09	1.00	1.19		1.02	0.90	1.16		1.09	1.00	1.18	
																
*Proportion of housing without bath or shower (%)*																
<1.1	1.00			0.113	1.00			0.104	1.00			0.147	1.00			0.664
1.1–1.7	1.00	0.94	1.05		1.01	0.93	1.09		0.98	0.87	1.10		1.02	0.94	1.10	
1.7–2.7	1.03	0.98	1.09		1.02	0.94	1.10		0.96	0.85	1.08		1.00	0.92	1.08	
>2.7	1.04	0.98	1.10		0.92	0.85	1.01		0.91	0.81	1.04		1.03	0.94	1.11	
																
*Proportion of manual workers (%)*																
<13	1.00			0.009	1.00			0.723	1.00			0.625	1.00			0.032
13–17	1.07	1.01	1.13		0.99	0.91	1.08		0.97	0.86	1.09		1.09	1.01	1.19	
17–21	1.10	1.04	1.16		1.03	0.95	1.12		0.96	0.86	1.08		1.11	1.03	1.20	
>21	1.07	1.01	1.14		0.97	0.89	1.06		0.97	0.86	1.09		1.10	1.00	1.19	

a 95% confidence interval.

RR=relative risk.
